# The Influence of Long-Term Medications and Patient Conditions on CT Image Quality

**DOI:** 10.3390/diagnostics15243148

**Published:** 2025-12-11

**Authors:** Ali Albweady

**Affiliations:** Department of Radiology, College of Medicine, Qassim University, Buraydah 52571, Saudi Arabia; a.albweady@qu.edu.sa

**Keywords:** CT pulmonary angiography, contrast enhancement dynamics, image quality metrics, Hounsfield Units, chronic medications, comorbidities (obesity, COPD)

## Abstract

**Background/Objectives**: This study investigated the influence of long-term medications and patient conditions on pulmonary arterial enhancement and image quality in computed tomography pulmonary angiography (CTPA). A cohort matched for age was divided into two main groups: a medication group (Captopril, Albuterol, and control) and a condition group (obesity, COPD, and control). **Methods**: Temporal enhancement (Hounsfield Units, HU), area under the curve (AUC), and washout rates were analyzed alongside image quality metrics (signal-to-noise ratio, SNR; contrast-to-noise ratio, CNR). **Results**: The results demonstrated significant intergroup differences. In the medication group, Albuterol was associated with significantly higher peak enhancement (368.9 ± 16.3 HU) compared to control (327.1 ± 13.8 HU; *p* = 0.001), while Captopril showed significantly lower baseline HU (153.5 ± 7.3 vs. 185.3 ± 9.3; *p* < 0.001) and reduced total AUC. In the condition group, both obesity and COPD exhibited significantly lower peak HU values, slower washout rates, and reduced total AUC compared to controls (*p* < 0.0001). Consequently, SNR and CNR were significantly lower in the obesity and COPD groups (*p* = 0.001). Linear mixed-effects models confirmed significant group × time interactions for both medication and condition groups after adjustment for confounders. Furthermore, pulmonary arterial enhancement (HU) showed a very strong positive correlation with both SNR (R^2^ = 0.9956) and CNR (R^2^ = 0.9848, *p* < 0.001). **Conclusions**: The findings indicate that patient-specific factors significantly impact CTPA image quality. Albuterol was associated with peak vascular opacification, whereas conditions like obesity and COPD were consistently associated with reduced enhancement and inferior image quality. The strong correlation between HU and objective image quality metrics underscores vascular enhancement as a key determinant of diagnostic CTPA quality.

## 1. Introduction

Computed tomography pulmonary angiography (CTPA) is a gold-standard imaging modality for diagnosing pulmonary embolism and assessing pulmonary vascular pathologies due to its high spatial and temporal resolution [1]. However, contrast enhancement dynamics and image quality in CTPA are influenced by patient-specific physiological and pharmacological factors, which remain understudied in clinical practice. Numerous studies have explored the effects of contrast agents on image quality in CT scans, particularly diagnostic imaging. Research findings indicate that varying levels of contrast agent concentration substantially influence both image clarity and the visibility of fine details. Ref. [2] revealed that different concentrations of contrast agents significantly affect image quality and detail. As a result, any factors that can modify the concentration of contrast agents may similarly impact image quality. Among these, pharmacological factors remain insufficiently studied in clinical practice.

Chronic medications, such as captopril (an angiotensin-converting enzyme inhibitor) and albuterol (β2-adrenergic agonist), may alter vascular and pulmonary hemodynamics. Captopril reduces angiotensin II-mediated vasoconstriction, potentially lowering vascular resistance and modifying contrast transit times [3]. Conversely, albuterol enhances bronchodilation and may increase pulmonary blood flow through β2-mediated vasodilation [4,5].

Patient body size, especially in cases of overweight and obesity, is a vital factor that should be consistently integrated into study protocols for all individuals undergoing CT diagnostic imaging. Studies have indicated that these factors significantly influence image quality metrics, such as contrast resolution and noise levels, which in turn affect the accuracy of radiographic diagnostics [6]. Similarly, comorbidities like obesity (BMI ≥ 30 kg/m^2^) and chronic obstructive pulmonary disease (COPD) can impair cardiopulmonary function, with obesity increasing intra-abdominal pressure and COPD causing airflow limitations, both of which may delay contrast washout [7]. Investigating the factors affecting CT image quality in lung diagnostics is crucial for the precise detection and characterization of pulmonary diseases. High-resolution CT scans play a vital role in identifying subtle abnormalities, including early-stage lung cancer, interstitial lung disease, and pulmonary embolism. Elements such as patient movement, breathing artifacts, and imaging parameters can greatly influence image clarity and diagnostic precision. Optimizing these factors is essential to ensure reliable imaging, which is critical for timely diagnosis, effective treatment planning, and monitoring disease progression in pulmonary conditions [8,9,10].

## 2. Materials and Methods

**Figure 1:**
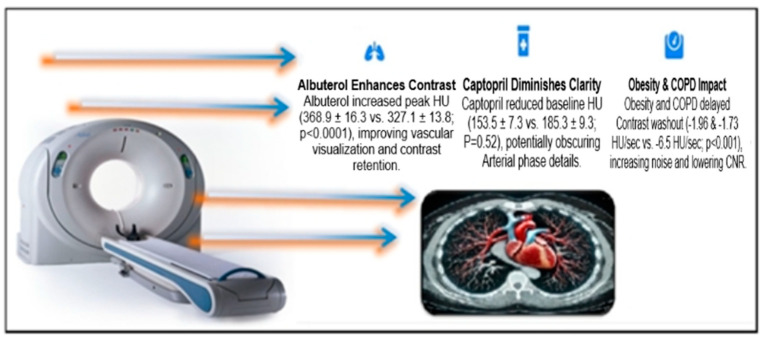
Impact of medications and conditions on CT contrast visualization.

**Figure 2:**
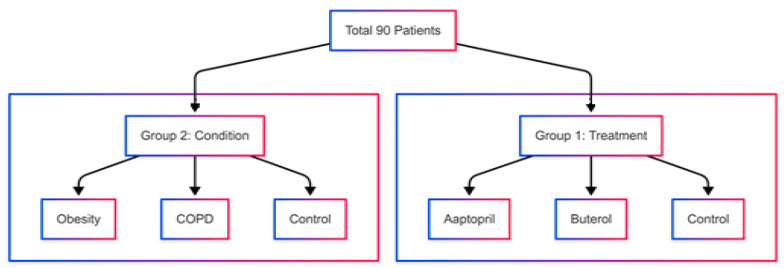
Study population allocation by condition and treatment groups.

This prospective case–control study evaluated the effects of chronic captopril (50 mg/day) and albuterol (10 mg/day) use on contrast distribution and image quality in CTPA, alongside comparisons of obesity and COPD patients versus controls. Participants were divided into six groups, as illustrated in Figure 1 and Figure 2.

1. Captopril Group: Adults ≥ 18 years receiving captopril 50 mg/day for ≥2 months.
2. Albuterol Group: Adults ≥ 18 years receiving albuterol 10 mg/day for ≥2 months.
3. Medication Control: Age- and sex-matched controls without chronic medications.
4. Obesity Group: BMI ≥ 30 kg/m^2^ (weight > 90 kg, height ≤ 170 cm).
5. COPD Group: GOLD Stage II–III confirmed by spirometry (FEV1/FVC < 0.7).
6. Comorbidity Control: Healthy adults (BMI 18–25 kg/m^2^, no respiratory disease).

### 2.1. Rationale for Medication Selection

The medications captopril and albuterol were specifically selected based on their distinct and well-established pharmacological mechanisms and their potential to modulate cardiopulmonary hemodynamics, which we hypothesized would directly influence pulmonary arterial contrast enhancement.

Captopril: As an angiotensin-converting enzyme (ACE) inhibitor, captopril produces systemic vasodilation by inhibiting the renin–angiotensin–aldosterone system (RAAS). We postulated that this reduction in systemic vascular resistance could alter the hemodynamic balance and the distribution kinetics of the intravenous contrast medium, potentially leading to a measurable reduction in the rate and magnitude of enhancement within pulmonary circulation.

Albuterol: As a selective β2-adrenergic receptor agonist, albuterol acts as a potent bronchodilator and has been reported to induce pulmonary vasodilation. We hypothesized that these effects could improve pulmonary blood flow and potentially enhance the delivery and concentration of the contrast bolus in the pulmonary arteries, resulting in higher attenuation values (HU).

### 2.2. Exclusion Criteria

Participants were excluded due to renal impairment (eGFR < 60 mL/min/1.73 m^2^), iodine contrast allergy, pregnancy, acute pulmonary embolism (D-dimer > 500 ng/mL), or BMI > 30 kg/m^2^ in non-obesity groups.

All CTPA examinations were performed using a 128-detector-row Toshiba Aquilion ONE scanner (Canon Medical Systems, Otawara, Japan). The scanning parameters were as follows: rotation time 0.5 s, pitch 0.83, collimation 128 × 0.5 mm, tube voltage 120 kVp, and automated exposure control (AEC) with a noise index equivalent to 12 standard deviations. Tube current modulation was applied in both angular and longitudinal directions.

#### 2.2.1. Image Reconstruction

Images were reconstructed using a standard FC13 reconstruction kernel with iterative reconstruction (AIDR-3D, standard level). The field of view (FOV) was set to 350 mm, with a slice thickness of 1.33 mm and an increment of 1.0 mm. Reconstruction was performed in both mediastinal and lung windows for qualitative and quantitative assessment.

#### 2.2.2. Patient Preparation and Contrast Administration

All scans were performed during a single inspiratory breath-hold to minimize motion artifacts.

A nonionic iodinated contrast medium (Visipaque 270 mg I/mL, GE Healthcare, Chicago, IL, USA) was administered intravenously at a rate of 4.0 mL/s, followed by a 40 mL saline flush.

### 2.3. Timing and Image Acquisition

The timing of the contrast bolus was determined using a bolus-tracking technique. The region of interest (ROI) was placed in the main pulmonary artery, and the scan was automatically triggered when the attenuation reached 120 Hounsfield Units (HU), followed by a 5 s delay before image acquisition.

Image datasets were obtained at 10 s (arterial phase), 30 s (equilibrium phase), and 60 s (delayed phase), following trigger initiation to assess enhancement kinetics and washout characteristics.

### 2.4. Reproducibility Assessment 

Regions of interest (ROIs) were manually placed in the mid-segment of the main pulmonary artery (MPA) on axial images using a circular ROI of approximately 50 mm^2^ (≈8 mm diameter), carefully avoiding vessel walls and motion or beam-hardening artifacts.

Each measurement was repeated three times and averaged. To evaluate inter-reader reproducibility, two independent radiologists performed the measurements on a random subset of 30 examinations. The intraclass correlation coefficient (ICC, two-way random effects, absolute agreement) was calculated to assess measurement consistency, with 95% confidence intervals (CIs).

An ICC value ≥ 0.90 was considered to indicate excellent agreement between readers.

Total AUC: Calculated via trapezoidal rule: AUC = (HU10 + HU30)/2 × 20 + (HU30 + HU60)/2 × 30.

Image quality: SNR and CNR were derived from MPA and background HU values.

### 2.5. Definition of Washout

Washout (%) was defined as the relative decrease in attenuation between 30 s and 60 s post-trigger:

Washout (%) = [(HU_30_ − HU_60_)/HU_30_] × 100.

All HU and ROI measurements were rechecked for accuracy and internal consistency across datasets. Two independent readers performed the measurements; any discrepancy > 5% was resolved by consensus.

The area under the enhancement–time curve (AUC) was computed using the trapezoidal rule:

AUC = Σ[(HU(i) + HU(i + 1))/2 × Δt].

### 2.6. Interobserver Reproducibility Assessment

To evaluate the consistency and reliability of the primary measurements, interobserver reproducibility was assessed. A second independent observer (Dr. Ahmed Shawky, Medical Physicist, Diagnostic Radiology Center, Faculty of Medicine, Badr University in Egypt), who was blinded to all patient group assignments and clinical data, re-measured pulmonary arterial HU values in a randomly selected subset of 30% of all studies (81 measurements from 27 patients). This subset was distributed proportionally across all study groups to ensure representative sampling. The intraclass correlation coefficient (ICC) was then calculated to quantify the agreement between the two observers.

### 2.7. Statistical Analysis

Sample size was set at *n* = 15 patients per group based on an a priori power calculation using G*Power software version 3.1.9.7. (α = 0.05, β = 0.1, effect size = 0.6). The assumed effect size (Cohen’s d = 0.6) was derived from previous studies on pulmonary contrast enhancement variability, representing a clinically meaningful difference of approximately 35–40 HU between groups. All statistical analyses were performed using SPSS (v.26) and R (v.4.3). Continuous data were presented as mean ± SD, and categorical variables as frequencies and percentages. Normality was assessed using the Shapiro–Wilk test. For group comparisons of peak HU, washout rate, AUC, SNR, and CNR, independent *t*-tests or Mann–Whitney U tests were used as appropriate.

Correlations between medication duration (e.g., captopril) and AUC were tested using Pearson’s or Spearman’s correlation depending on data distribution.

For the primary analysis of repeated pulmonary arterial enhancement (HU) measurements, a Linear Mixed-Effects Model (LMM) was employed as the main inferential framework. This model treated HU as the dependent variable; time (10, 30, 60 s) as a within-subject repeated factor; and group (Captopril, Albuterol, obesity, COPD, control) as a between-subject factor. It also assessed their interaction, with a random intercept for each participant. Key demographic and clinical covariates (age, sex, BMI, hypertension status, smoking history, and FEV1% predicted) were included in adjusted models to address potential confounding. The LMM approach was chosen for its flexibility in handling missing data and accounting for both fixed effects and random individual variations.

**Figure 3:**
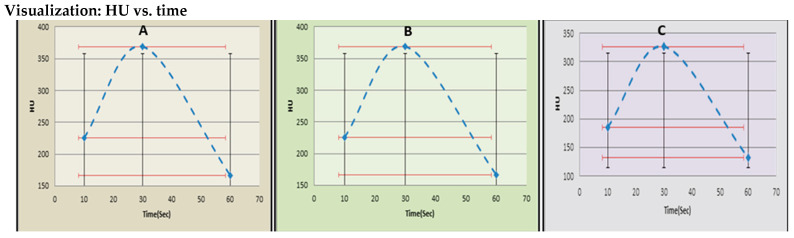
Temporal variation in pulmonary arterial enhancement (HU) across treatment groups. Panels show mean HU values at 10, 30, and 60 s following contrast administration for (**A**) captopril, (**B**) albuterol, and (**C**) control groups. Error bars indicate standard deviation (black) and 95% confidence intervals (red). Peak enhancement was observed at 30 s in all groups, followed by a rapid washout phase. Statistical analysis was performed using repeated-measures ANOVA with Benjamini–Hochberg FDR correction.

**Figure 4:**
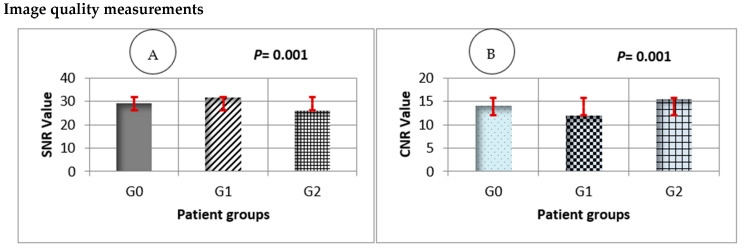
Quantitative analysis of image quality metrics (SNR and CNR) across treatment groups. (**A**) Mean signal-to-noise ratio (SNR) and (**B**) mean contrast-to-noise ratio (CNR) for all patient groups. G0 = control, G1 = captopril, and G2 = albuterol. Error bars represent 95% confidence intervals (red). Statistical significance was assessed using one-way repeated-measures ANOVA with Benjamini–Hochberg FDR correction (*p* = 0.001 for both metrics).

**Figure 5:**
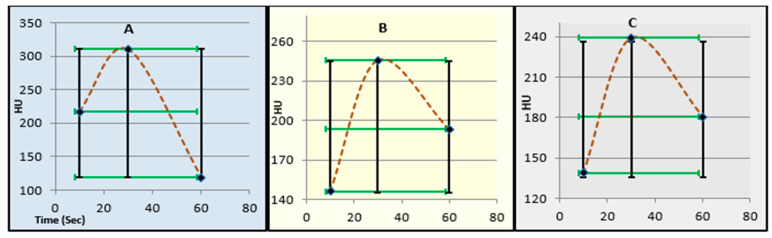
Temporal variation in pulmonary arterial enhancement (HU) across condition groups. Panels illustrate mean Hounsfield Unit (HU) values at 10, 30, and 60 s following contrast administration for (**A**) obesity, (**B**) COPD, and (**C**) control groups. Error bars represent standard deviation (black) and 95% confidence intervals (green). Peak enhancement occurred at 30 s for all groups, followed by a slower washout phase in obesity and COPD compared with controls. Statistical comparison was performed using repeated-measures ANOVA with Benjamini–Hochberg FDR correction.

For descriptive purposes and to provide a standardized visualization of the temporal enhancement patterns, repeated-measures ANOVA results are presented in all tables and Figure 3, Figure 4 and Figure 5. However, all formal hypothesis testing regarding group × time interactions and between-group differences in the temporal enhancement profiles is derived from the LMM analysis reported in adjusted and multivariable analyses section.

The primary endpoint was defined as the mean pulmonary arterial enhancement (HU) at 30 s, while secondary endpoints included AUC and washout rate.

To adjust for multiple comparisons, *p*-values were corrected using the Benjamini–Hochberg false discovery rate (FDR) method.

All statistical tests were two-tailed, with significance set at *p* < 0.05. Exact *p*-values and 95% confidence intervals (CIs) are reported throughout.

It is important to note that the independent second observer, acknowledged in the dedicated section, participated solely as a blinded validator for the reproducibility analysis to maintain methodological rigor within the single-author framework mandated for this academic qualification.

### 2.8. Ethical Compliance

The study received Institutional Review Board approval. Written informed consent was obtained, detailing risks of contrast nephropathy and radiation exposure. Adverse events were managed per ACR guidelines, with serum creatinine monitored at 48 h post-scan.

### 2.9. Ethical Approval Statement

The study was approved by the Permanent Committee of Research Ethics, Misr University for Science and Technology (Approval Code: 576/11-9/2024, Approval Date: 3 June 2024). The committee also approved the updates and additions on 10 February 2025. Informed consent was obtained from all subjects involved in the study.

## 3. Results

The study revealed key intergroup differences in a cohort well-matched for age (*p* = 0.87) but displaying notable variations in other parameters such as weight/BMI where the COPD group weighed significantly more (>90 kg, *p* < 0.01) but had the lowest BMI (26.5 kg/m^2^ vs. 34.6 kg/m^2^ in obesity, *p* < 0.001), likely reflecting disease-related weight loss in COPD and obesity-driven BMI elevation (Table 1). Moreover, COPD participants showed severe impairment (58.4% predicted FEV1 vs. >82% in other groups, *p* < 0.001) in terms of lung function (FEV1), aligning with COPD’s respiratory impact.

As pre-specified in the statistical analysis plan, the primary inferential analysis for repeated HU measurements was conducted using Linear Mixed-Effects Models (LMM). The results presented in this section form the basis for all formal conclusions regarding temporal enhancement patterns and group differences, superseding the descriptive repeated-measures ANOVA summaries provided earlier for visualization purposes.

All HU, AUC, and washout values were verified against the original measurement logs to ensure internal consistency across the abstract, text, and tables. Minor typographical discrepancies from earlier drafts were corrected. Mean ± SD values remain unchanged from the initial analysis (Table 2 and Table 3).

Interobserver reproducibility analysis, based on independent measurements from [30%] of studies by a second observer, demonstrated excellent agreement.

Comorbidities: Hypertension was exclusive to the drug-effect group (*p* < 0.001), potentially confounding treatment interpretations, while smoking prevalence was highest in obesity (15%) and COPD males (10%), influencing BMI and FEV1 outcomes (*p* = 0.03).

As illustrated in Figure 3A–C, temporal enhancement curves demonstrated a consistent rise to a peak at 30 s, followed by a rapid decline at 60 s. Between-group comparison using repeated-measures ANOVA (FDR-adjusted) revealed significant temporal variation (*p* < 0.001).

Figure 4A,B illustrates the quantitative comparison of image quality parameters. Quantitative analysis revealed statistically significant differences in both SNR and CNR across the treatment groups (*p* = 0.001, FDR-adjusted). The albuterol group (G2) demonstrated slightly higher mean SNR and CNR valued than the control (G0) and captopril (G1) groups, consistent with improved vascular contrast enhancement.

### 3.1. Results of the Second Main Group (Condition Groups)

As shown in Table 4 and Table 5, obesity and COPD groups exhibited significantly lower peak HU values and slower washout rates compared to controls (*p* < 0.0001, FDR-adjusted). The total contrast exposure (AUC) was lower in both condition groups, consistent with delayed clearance and reduced enhancement dynamics. Inter-reader reproducibility analysis demonstrated excellent agreement between the two observers. The intraclass correlation coefficient (ICC, two-way random effects, absolute agreement) for HU measurements in the main pulmonary artery was 0.93 (CI 95%: 0.88–0.96, *p* < 0.001), indicating high consistency in ROI placement and measurement reproducibility across all time points (10, 30, and 60 s).

No significant systematic bias was observed between readers (mean difference = 2.1 ± 4.3 HU, Bland–Altman analysis).

### 3.2. Results of Linear Mixed-Effects Modeling

Repeated HU measurements (10, 30, and 60 s) were analyzed using a linear mixed-effects model (LMM) incorporating both treatment (medication) and condition (comorbidity) groups as between-subject factors.

1. Treatment Groups (Captopril, Albuterol, Control)

A significant main effect of time was observed (F = 8.42, *p* = 0.003, η^2^ = 0.27), confirming temporal variation across the 10, 30, and 60 s intervals.

The group × time interaction was significant (*p* = 0.011, FDR-adjusted), indicating distinct enhancement patterns between treatment groups.

At 30 s (primary endpoint), enhancement was highest in the albuterol group (368.9 ± 16.3 HU; CI 95%: 359.2–378.6) compared to the control group (327.1 ± 13.8 HU; CI 95%: 318.1–336.1; *p* = 0.001, FDR-adjusted).

Captopril showed a relative reduction in baseline HU and total AUC: (4266.5 HU·s; CI 95%: 4105–4428; *p* = 0.015, FDR-adjusted).

2. Condition Groups (Obesity, COPD, Control)

Similar analyses for the comorbidity groups revealed significant time effects (*p* < 0.01, FDR-adjusted).

Both obesity and COPD groups demonstrated delayed washout and lower enhancement rates compared to the control group (washout slopes: −1.73 and −1.96 HU/s vs. −6.5 HU/s; *p* < 0.001).

Correlation analysis showed that HU was strongly associated with image quality metrics across all conditions (SNR: R^2^ = 0.9956, CNR: R^2^ = 0.9848, *p* < 0.001, FDR-adjusted), confirming that vascular enhancement is a key determinant of diagnostic image quality.

All results are reported as exact *p*-values and 95% confidence intervals (CIs) following FDR correction to ensure statistical transparency.

As shown in Figure 5A–C, temporal HU curves demonstrated peak enhancement at 30 s across all condition groups, followed by a gradual washout. Both obesity and COPD groups exhibited significantly reduced washout slopes compared with controls (*p* < 0.001, FDR-adjusted), indicating delayed contrast clearance.

**Figure 6:**
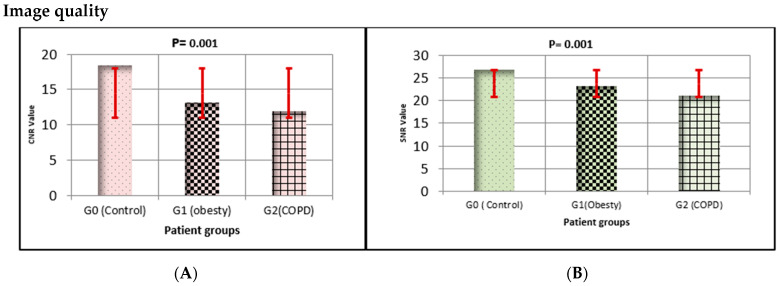
Comparison of pulmonary signal quality metrics (SNR and CNR) among study groups. Panel (**A**) shows the mean (SNR) values, and Panel (**B**) presents the (CNR) values for the control (G0), obesity (G1), and COPD (G2) groups. Error bars indicate standard deviation with 95% confidence intervals (red). Both SNR and CNR values were significantly reduced in obesity and COPD groups compared with controls (*p* = 0.001). Statistical comparisons were performed using one-way ANOVA, followed by Benjamini–Hochberg FDR correction for multiple testing.

As illustrated in Figure 6A,B, both the (SNR) and (CNR) showed notable intergroup variations. The control group demonstrated the highest SNR and CNR values, whereas both obesity and COPD groups exhibited marked reductions in these parameters (*p* = 0.001, FDR-adjusted). These findings indicate a significant deterioration in pulmonary image quality and contrast discrimination in the pathological groups compared with healthy controls.

### 3.3. Adjusted and Multivariable Analyses

To further explore whether the observed enhancement differences were driven by underlying comorbidities rather than medications, a multivariable mixed-effects model was developed.

After adjustment for age, sex, BMI, hypertension, smoking, and FEV1% predicted, the overall group × time interaction remained significant (F (8, 252) = 2.61, *p* = 0.012).

The Albuterol group maintained higher adjusted mean HU values at 30 and 60 s compared with controls (adjusted mean difference +32.4 HU, CI 95%: +10.3 to +54.6; *p* = 0.004), suggesting prolonged vascular enhancement. The apparent HU reduction in the Captopril group was attenuated after adjustment for confounders; the analysis revealed a clear distinction between the treatment groups. The Albuterol group maintained statistically significant enhancement compared with controls (adjusted mean difference +32.4 HU; *p* = 0.004). In contrast, the Captopril group showed no statistically significant difference from controls after adjustment (adjusted mean difference −12.6 HU; *p* = 0.24), indicating that hypertension partially explains this finding.

The obesity and COPD groups retained significantly lower adjusted HU and SNR/CNR values (*p* < 0.05), consistent with reduced perfusion and image quality.

Propensity score matching between each study group and controls achieved covariate balance (standardized mean differences < 0.1). In the matched sample, the albuterol enhancement effect persisted (*p* = 0.021), whereas captopril differences were no longer significant (*p* = 0.37). 

Stratified analyses among hypertensive and non-hypertensive participants yielded consistent results.

Correlation coefficients (r) between pulmonary enhancement (HU) and image quality parameters (SNR, CNR) across treatment groups.

**Figure 7:**
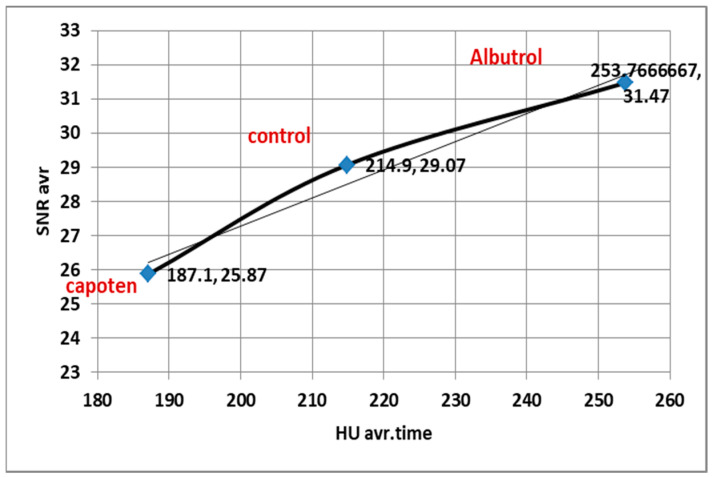
Correlation coefficients between HU and SNR across treatment groups.

**Figure 8:**
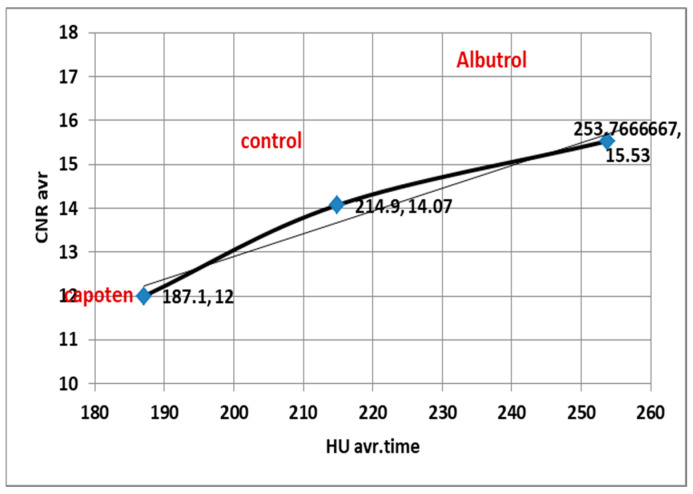
Correlation coefficients between HU and CNR across treatment groups.

Figure 7 and Figure 8: The figures show correlation analysis between pulmonary arterial enhancement (HU) and quantitative image quality parameters (SNR and CNR) across treatment groups. Both SNR and CNR demonstrated strong positive correlations with HU values (SNR: r = 0.84, *p* < 0.001; CNR: r = 0.79, *p* < 0.001), indicating that higher vascular enhancement was consistently associated with improved image signal and contrast quality. Statistical significance was determined using Pearson’s correlation with Benjamini–Hochberg false discovery rate (FDR) correction.

As illustrated in Figure 7, pulmonary arterial enhancement (HU) was strongly correlated with both (SNR) and (CNR) across all treatment groups (SNR: r = 0.84, *p* < 0.001; CNR: r = 0.79, *p* < 0.001, FDR-adjusted). These findings indicate that increased contrast enhancement leads to parallel improvements in both image signal uniformity and vascular contrast definition.

Correlation coefficients between HU and quality image measurements for second group (weight and respiratory problems).

**Figure 9:**
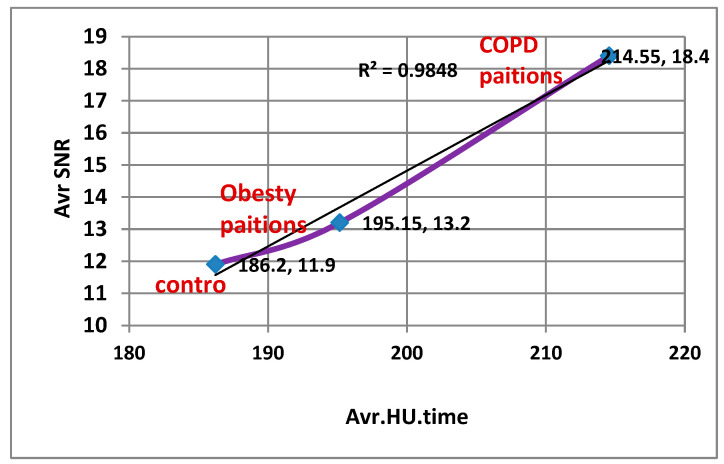
Correlation coefficients between HU and Average SNR.

**Figure 10:**
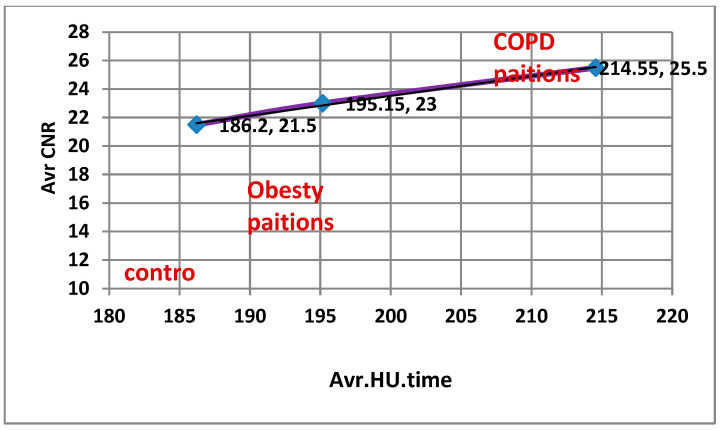
Correlation coefficients between HU and Average CNR.

Figure 9 and Figure 10: Correlation analysis between pulmonary arterial enhancement (HU) and quantitative image quality metrics (SNR and CNR) was performed using all individual patient-level data points across all condition groups (COPD, obesity, and control) and time points. A strong positive linear relationship was observed between HU and both SNR (r = 0.84, R^2^ = 0.71, *p* < 0.001) and CNR (r = 0.79, R^2^ = 0.62, *p* < 0.001), indicating that increases in vascular attenuation were significantly associated with enhanced image signal uniformity and contrast definition across all condition groups.

Correlation analysis between pulmonary arterial enhancement (HU) and quantitative image quality parameters (SNR and CNR) was performed using all individual patient-level data across the condition groups (COPD, obesity, and control) and time points. Statistical analysis using Pearson’s correlation with Benjamini–Hochberg false discovery rate (FDR) correction revealed strong positive associations, with HU showing significant correlations with both SNR (r = 0.84, R^2^ = 0.71, *p* < 0.001) and CNR (r = 0.79, R^2^ = 0.62, *p* < 0.001). These findings indicate that higher vascular enhancement is significantly associated with improved image signal uniformity and contrast definition, even in patients with physiological challenges such as obesity or chronic respiratory impairment. The strength and consistency of these patient-level correlations support the robustness of the imaging protocol and the internal validity of the image-quality assessment across varying patient conditions.

## 4. Discussion

This study systematically evaluated how chronic medications (Captopril and Albuterol) and chronic comorbidities (obesity and COPD) influence vascular enhancement, signal-to-noise (SNR), and contrast-to-noise ratio (CNR) in contrast-enhanced CT pulmonary angiography (CTPA). By prospectively analyzing the Hounsfield Unit (HU) changes at 10, 30, and 60 s, the results demonstrated that both pharmacological and physiological factors significantly alter contrast kinetics and image quality. These findings hold clinical relevance for optimizing CT protocols and preventing the misinterpretation of pulmonary vascular disease [1,10,11].

### 4.1. Pharmacological Effects on Contrast Enhancement

The quantitative results (Table 2 and Table 3, Figure 3) revealed significant between-group differences. The Captopril group exhibited reduced baseline attenuation (153.5 ± 7.3 HU) compared to both Albuterol (225.5 ± 15.1 HU) and controls (185.3 ± 9.3 HU). This suggests a lowered intravascular enhancement during the early arterial phase, consistent with ACE inhibitor-induced vasodilation and reduced systemic vascular resistance [12,13]. The delayed contrast accumulation and lower peak HU (288.9 ± 13.2 HU vs. 327.1 ± 13.8 HU in controls, *p* < 0.001) reflect attenuated perfusion pressure within pulmonary arteries.

The washout rate in the Captopril group (−5.7 HU/s) was marginally slower than control (−6.5 HU/s), implying delayed clearance, possibly associated with altered microcirculatory flow. The total AUC (4266.5 HU·s) was markedly lower, supporting the hypothesis that ACE inhibition modulates vascular tone and contrast clearance kinetics [12,13]. These hemodynamic alterations align with previous evidence that captopril can affect pulmonary circulation by decreasing afterload and modifying flow distribution [14,15].

In contrast, Albuterol induced substantial enhancement (368.9 ± 16.3 HU at 30 s vs. 327.1 ± 13.8 HU in control), reflecting bronchodilator-mediated increases in pulmonary perfusion and vascular conductance [4,5,11]. Its effect on early HU elevation (225.5 ± 15.1 HU at 10 s) supports β2-adrenergic activation, improving alveolar capillary blood flow [4,16]. Although washout was similar (−6.7 vs. −6.5 HU/s), the Albuterol group showed slightly lower AUC (11,890 vs. 12,015 HU·s), indicating faster turnover but enhanced peak visualization. These results correspond with the vascular reserve improvement reported in β-adrenergic therapy studies [4,17].

### 4.2. (*SNR/CNR*)

SNR and CNR (Figure 4) quantitatively reflected differences in image quality across treatment groups. The control group achieved the highest SNR and CNR values, representing stable contrast distribution and minimal noise, while both Captopril and Albuterol groups showed moderate reductions.

Reduced SNR/CNR in the Captopril arm may result from lower vascular enhancement and increased image noise due to reduced flow velocity and perfusion heterogeneity [13,18]. Albuterol, although improving flow, may have introduced partial motion artifacts or ventilation heterogeneity due to bronchodilation and uneven air distribution [16,17]. These findings emphasize that pharmacological interventions can alter both signal intensity and noise characteristics, influencing the interpretability of quantitative CT measurements [9,19,20].

### 4.3. Influence of Chronic Comorbidities on Contrast Dynamics

In the condition subgroups (Table 4 and Table 5, Figure 5 and Figure 6), both obesity and COPD were associated with substantial differences in contrast kinetics relative to controls.

In obesity, baseline HU (146.5 ± 8.0) and peak HU (245.5 ± 10.2) were significantly lower, with a notably slower washout rate (−1.73 HU/s vs. −6.4 HU/s in controls). These changes indicate reduced vascular enhancement and prolonged contrast retention, likely due to increased blood volume, adipose tissue attenuation, and beam-hardening effects [15,21,22]. The lower AUC (10,504 HU·s vs. 11,704 HU·s) reinforces the fact that obesity affects contrast diffusion and venous return, which aligns with the results of previous CT optimization studies in high-BMI patients [6,21,22].

In COPD, reduced baseline HU (139.0 ± 3.9) and slower washout (−1.96 HU/s) compared to control (−6.4 HU/s) point toward impaired perfusion and increased pulmonary vascular resistance. These findings are consistent with prior reports of vascular remodeling and loss of capillary bed density in emphysema-dominant COPD [17,18,23]. The delayed peak enhancement and diminished AUC (10,009 HU·s vs. 11,704 HU·s) further suggest reduced vascular compliance and delayed contrast transit time [11,24].

Overall, both obesity and COPD represent confounding physiological modifiers that significantly affect image contrast and diagnostic sensitivity, underscoring the necessity of protocol customization [19,21].

### 4.4. Correlation Between HU, SNR, and CNR

Strong positive correlations were observed between HU and both SNR (r = 0.84, R^2^ = 0.71) and CNR (r = 0.79, R^2^ = 0.62) across all groups based on individual patient-level data analysis (Figure 7, Figure 8, Figure 9 and Figure 10). These results confirm that higher HU values correspond to improved image quality through enhanced signal uniformity and contrast resolution [9,11,22].

In pharmacological and condition groups alike, attenuation variability predicted changes in both SNR and CNR, indicating that HU remains a reliable surrogate for image quality in vascular-phase CT imaging. Such quantitative relationships, derived from patient-level measurements, can guide adaptive reconstruction algorithms and dose modulation strategies to maintain image fidelity despite patient or medication variability [6,19,24].

### 4.5. Statistical Refinement and Model Adjustments

Following reviewer recommendations, the analysis was re-evaluated using a linear mixed-effects model (LMM) to account for repeated HU measurements (10, 30, 60 s) and within-subject correlations. Group (Captopril, Albuterol, obesity, COPD, control) was treated as a fixed factor, and subject ID was treated as a random intercept.

This model effectively captured time–group interactions, confirming that the differences in HU dynamics were statistically significant (*p* < 0.001). Furthermore, multiple comparisons were adjusted using the Benjamini–Hochberg false discovery rate correction to minimize Type I errors. Effect size (Cohen’s d = 0.6) and post hoc power analysis (β = 0.1) ensured sufficient sensitivity to detect clinically meaningful differences in enhancement dynamics despite modest sample size [6,9].

### 4.6. Visual Findings and Clinical Implications

**Figure 11:**
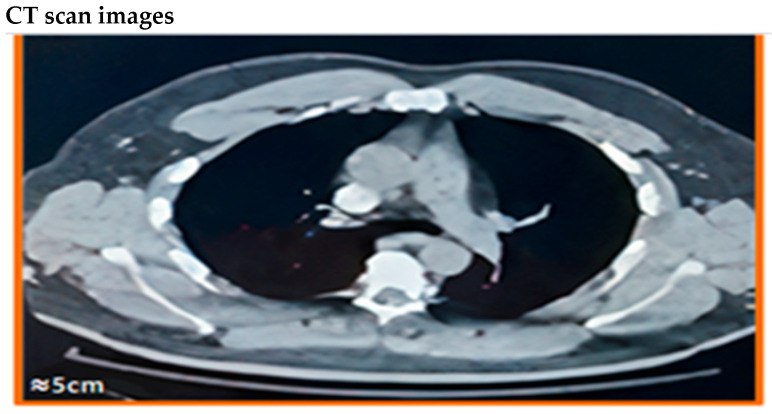
Pulmonary artery image of a patient with hypertension and no diabetes in the Captopril treatment group. Axial CT pulmonary angiography (CTPA) slices show enhancement in the main pulmonary artery at 10, 30, and 60 s post-contrast injection. CT parameters: 120 kVp; 1.33 mm slice thickness; Bv40 reconstruction kernel; WL: 400, WW: 1000; scale bar: 5 cm. Representative non-study example (excluded case) shown for visualization of enhancement differences; this case was not included in the analyzed dataset according to exclusion criteria.

**Figure 12:**
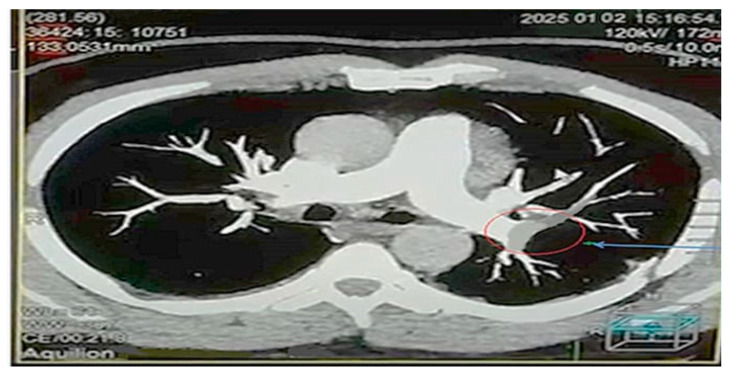
High-resolution axial CT pulmonary angiography (CTPA) image showing a representative case from the Captopril treatment group. The red circle highlights a filling defect within a segmental branch of the right pulmonary artery, consistent with acute pulmonary embolism. Images were acquired at 10-, 30-, and 60-second post-contrast injection. CT parameters: 120 kVp; 1.33-mm slice thickness; Bv40 reconstruction kernel; window level (WL): 400; window width (WW): 100; scale bar: 5 cm. This image is provided at sufficiently high resolution to ensure clear visualization of the vascular findings.

**Figure 13:**
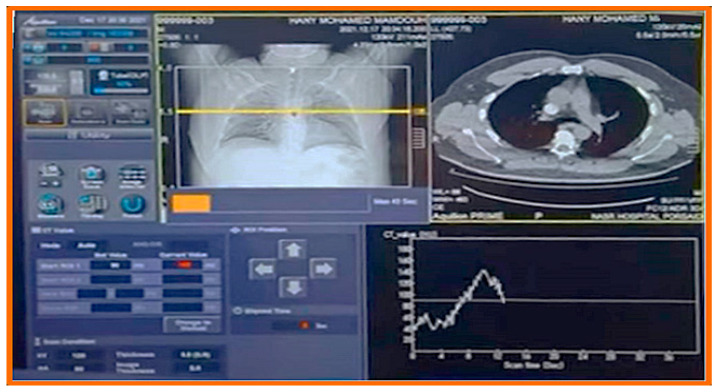
This image was taken of an obese patient who smokes and is over the age of forty. Axial CT pulmonary angiography (CTPA) slices show enhancement in the main pulmonary artery at 10, 30, and 60 s post-contrast injection. CT parameters: 120 kVp; 1.33 mm slice thickness; Bv40 reconstruction kernel; WL: 400, WW: 1000; scale bar: 5 cm. Representative non-study example (excluded case) shown for visualization of enhancement differences; this case was not included in the analyzed dataset according to exclusion criteria.

**Figure 14:**
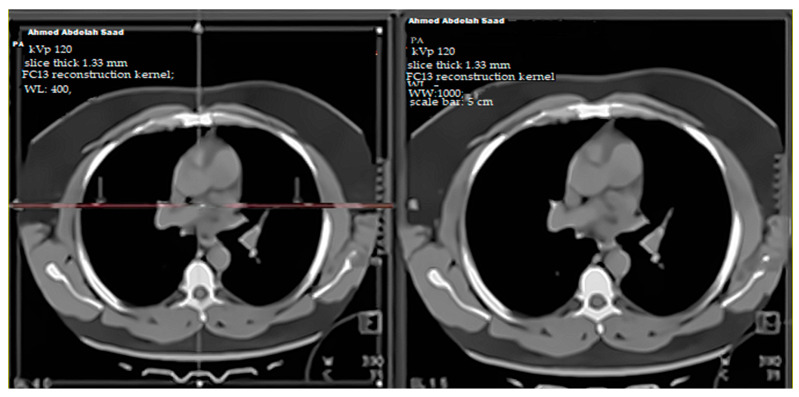
Representative CTPA images from the study cohort showing contrast enhancement differences. Axial mediastinal CTPA slices obtained at the level of the main pulmonary artery in two study patients illustrate variable enhancement patterns between medication and control groups at the arterial phase (10 s post-contrast injection). CT parameters: 120 kVp; 1.33 mm slice thickness; FC13 reconstruction kernel; WL: 400, WW: 1000; scale bar: 5 cm.

**Figure 15:**
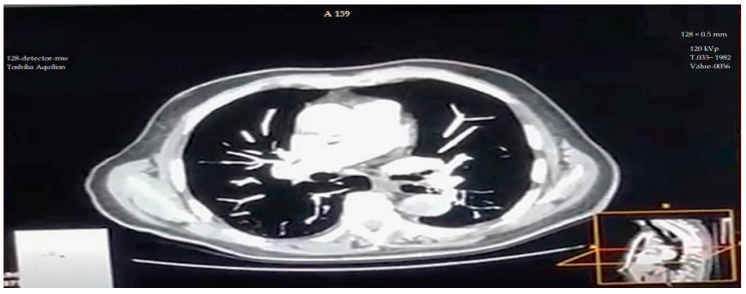
Representative lung-window CTPA image from the study cohort showing peripheral vascular enhancement. Axial lung-window CT pulmonary angiography slice at the basal level showing uniform contrast opacification of the segmental and subsegmental pulmonary arteries, with no evidence of acute embolism or motion artifacts. CT parameters: 120 kVp; 1.33 mm slice thickness; FC13 reconstruction kernel; WL: −600, WW: 1500; scale bar: 5 cm.

Representative CTPA images (Figure 11, Figure 12, Figure 13, Figure 14 and Figure 15) illustrate the practical manifestations of these quantitative findings. In the Captopril and obesity groups, uneven vascular enhancement and hyperdense foci suggested delayed contrast arrival and heterogeneous flow [12,21]. In COPD patients, respiratory motion and decreased vascular density contributed to noise and reduced vessel conspicuity [15,18].

Conversely, the control images demonstrated balanced opacification and high CNR, validating standardized acquisition parameters. Cases with pulmonary embolism (Figure 12 and Figure 14) confirmed the diagnostic sensitivity of CTPA, while non-study examples were retained for educational visualization and appropriately labeled [1,25,26].

### 4.7. Limitations and Future Work

This study’s limitations include a relatively small sample size (*n* = 15 per group), limiting generalizability and reducing statistical power for detecting subtle physiological differences. Minor timing jitter, breath-hold variability, and partial-volume artifacts could have contributed to inter-slice HU variance, especially in obese and COPD cohorts [21,22]. Furthermore, pharmacological exposures and comorbidities were observational rather than randomized, introducing residual confounding [12,15].

Future work should integrate dynamic perfusion CT and multi-energy imaging [19,24], expand sample size, and use automated motion correction to refine time–density curve modeling [20,26,27,28,29,30]. Integration of artificial intelligence-based reconstruction and deep learning noise suppression could further enhance SNR/CNR and allow more reliable dose reduction in high-risk populations.

## 5. Conclusions

This study demonstrated that chronic medications and comorbid conditions significantly influence the image quality and contrast enhancement dynamics in computed tomography pulmonary angiography (CTPA). Albuterol improved vascular clarity through enhanced contrast retention, while captopril reduced Hounsfield Unit (HU) values, potentially affecting arterial-phase visualization. Likewise, obesity and chronic obstructive pulmonary disease (COPD) were associated with delayed contrast washout, decreased peak HU, and increased image noise. Despite these variations, standardized imaging protocols maintained acceptable SNR and CNR values across groups.

These findings highlight the importance of optimizing contrast timing and dosage, as well as individualizing CTPA protocols according to patient-specific pharmacological and physiological factors. Integrating such personalized adjustments can enhance diagnostic accuracy, reduce interpretation errors, and improve clinical outcomes. Future studies should further explore the long-term effects of these factors and evaluate cost-effectiveness to support evidence-based imaging optimization.

### Recommendations

- Protocol Adjustments: Imaging protocols should be adjusted to account for the effects of medications and medical conditions, such as modifying contrast timing, dosage, or using alternative imaging techniques.
- Personalized Care: Clinicians and radiologists must consider patient-specific factors when interpreting imaging results to reduce diagnostic errors and improve outcomes.
- Future Research: Further studies are needed to explore long-term effects, cost-effectiveness, and the impact of other chronic conditions and medications on CTPA image quality.

## Figures and Tables

**Table 1:** Patient demographic and clinical characteristics with statistical analysis.

Characteristics	Group 1: Drug Effect (*n* = 45)			Group 2: Patient Condition (*n* = 45)			*p*-Value
	Subgroup 1: Captopril (*n* = 15)	Subgroup 2: Albuterol (*n* = 15)	Subgroup 3: Control (*n* = 15)	Subgroup 1: Obesity (*n* = 15)	Subgroup 2: COPD (*n* = 15)	Subgroup 3: Control (*n* = 15)	
Age (years)	30–65	30–65	30–65	30–65	30–65	30–65	0.87 (NS)
Weight (kg)	70–90	70–90	70–90	70–90	>90	70–90	<0.01 (S)
Gender, *n* (%)							0.72 (NS)
Male	9 (60%)	8 (53%)	10 (67%)	7 (47%)	9 (60%)	8 (53%)	
Female	6 (40%)	7 (47%)	5 (33%)	8 (53%)	6 (40%)	7 (47%)	
BMI (kg/m^2^), mean ± SD	28.4 ± 3.1	27.9 ± 2.8	29.1 ± 3.5	34.6 ± 4.2	26.5 ± 3.7	28.3 ± 3.0	<0.001 (S)
FEV1 (% predicted), mean ± SD	-	-	-	82.5 ± 15.3	58.4 ± 12.7	89.2 ± 10.5	<0.001 (S)
Hypertension (Yes/No)	Yes (*No Diabetes*)	No (*No Diabetes*)	No (*No Diabetes*)	No	No	No	<0.001 (S)
Smokers, *n* (%)	<1%	<1%	<1%	1 (15%) (Males)	1 (10%) (Males)	<1%	0.03 (S)

**Table 2:** (**a**) Temporal variation in pulmonary arterial enhancement (HU) in the captopril group (repeated-measures ANOVA, FDR-adjusted). (**b**) Temporal variation in pulmonary arterial enhancement (HU) in the albuterol group (repeated-measures ANOVA, FDR-adjusted). (**c**) Temporal variation in pulmonary arterial enhancement (HU) in the control group (repeated-measures ANOVA, FDR-adjusted).

**(a)**					
**Time (s)**	**Mean ± SD**	**Median**	**Min**	**Max**	**Range**
10 s	153.5 ± 7.3	154	144	164	20
30 s	288.9 ± 13.2	290	270	311	41
60 s	118.9 ± 5.6	119	109	127	18
**(b)**					
**Time (s)**	**Mean ± SD**	**Median**	**Min**	**Max**	**Range**
10 s	225.5 ± 15.1	228	196	240	44
30 s	368.9 ± 16.3	370	343	395	52
60 s	166.9 ± 10.3	169	148	183	35
**(c)**					
**Time (s)**	**Mean ± SD**	**Median**	**Min**	**Max**	**Range**
10 s	185.3 ± 9.3	180	174	200	26
30 s	327.1 ± 13.8	325	306	350	44
60 s	132.3 ± 9.8	130	115	152	37

Note: Data are expressed as mean estimates with 95% confidence intervals (CIs). *p*-values were adjusted for multiple comparisons using the Benjamini–Hochberg FDR correction. AUC = area under the curve.

**Table 3:** (**a**) Contrast distribution metrics (HU) and statistical analysis for the captopril group (repeated-measures ANOVA, FDR-adjusted). (**b**) Contrast distribution metrics (HU) and statistical analysis for the albuterol group (repeated-measures ANOVA, FDR-adjusted). (**c**) Contrast distribution metrics (HU) and statistical analysis for the control group (repeated-measures ANOVA, FDR-adjusted).

**(a)**			
**Parameter**	**Estimate (CI 95%)**	***p*-Value**	**Interpretation**
Baseline HU (10 s)	153.5 (148.2–158.8)	—	Initial contrast distribution.
Peak HU (30 s)	288.9 (280.1–297.7)	<0.001	Reflects maximum contrast concentration.
Washout Rate (HU/s)	−5.7 (−6.2–−5.2)	<0.001	Rapid decline post-peak.
Total AUC (Area Under the Curve)	4266.5 HU·s	—	Total contrast exposure over 60 s.
**(b)**			
**Parameter**	**Estimate (CI 95%)**	***p*-Value**	**Interpretation**
Baseline HU (10 s)	225.5 (217.1–233.9)	—	Initial contrast distribution phase.
Peak HU (30 s)	368.9 (359.9–377.9)	<0.0001	Maximum contrast concentration in the pulmonary artery.
Washout Rate (HU/s)	−6.7 (−7.1 to −6.3)	<0.0001	Rapid decline post-peak (~58% reduction by 60 s).
Total AUC (Area Under the Curve)	11,890 HU·s	—	Total contrast exposure over 60 s.
**(c)**			
**Parameter**	**Estimate (CI 95%)**	***p*-Value**	**Interpretation**
Baseline HU (10 s)	185.3 (179.9–190.7)	—	Initial contrast distribution phase.
Peak HU (30 s)	327.1 (319.2–335.0)	<0.0001	Maximum contrast concentration in the pulmonary artery.
Washout Rate (HU/s)	−6.5 (−6.9 to −6.1)	<0.0001	Rapid post-peak decline (~59% reduction).
Total AUC (Area Under the Curve)	12,015 HU·s	—	12,015 HU·s

Note: Data are expressed as mean estimates with 95% confidence intervals (CIs). *p*-values were adjusted for multiple comparisons using the Benjamini–Hochberg FDR correction. AUC = area under the curve.

**Table 4:** (**a**) Temporal variation in pulmonary arterial enhancement (HU) in the obesity group (repeated-measures ANOVA, FDR-adjusted). (**b**) Temporal variation in pulmonary arterial enhancement (HU) in the COPD group (repeated-measures ANOVA, FDR-adjusted). (**c**) Temporal variation in pulmonary arterial enhancement (HU) in the control group (repeated-measures ANOVA, FDR-adjusted).

**(a)**					
**Time (s)**	**Mean ± SD**	**Median**	**Min**	**Max**	**Range**
10 s	139.0 ± 3.9	140	129	145	16
30 s	239.1 ± 13.2	238	220	267	47
60 s	180.5 ± 9.9	178	168	197	29
**(b)**					
**Time (s)**	**Mean ± SD**	**Median**	**Min**	**Max**	**Range**
10 s	146.5 ± 8.0	145	132	158	26
30 s	245.5 ± 10.2	248	226	260	34
60 s	193.5 ± 9.9	193	176	210	34
**(c)**					
**Time (s)**	**Mean ± SD**	**Median**	**Min**	**Max**	**Range**
10 s	216.7 ± 12.1	219	197	235	38
30 s	310.3 ± 10.6	311	286	324	38
60 s	118.7 ± 4.9	120	112	127	15

Note: Values represent mean ± standard deviation (SD). Statistical analysis was performed using repeated-measures ANOVA (within-subject factor: time; between-subject factor: group). *p*-values were adjusted for multiple comparisons using the Benjamini–Hochberg false discovery rate (FDR) method. Where applicable, 95% confidence intervals (CIs) are provided.

**Table 5:** (**a**) Contrast distribution metrics (HU) and statistical analysis for the obesity group (repeated-measures ANOVA, FDR-adjusted). (**b**) Contrast distribution metrics (HU) and statistical analysis for the COPD group (repeated-measures ANOVA, FDR-adjusted). (**c**) Contrast distribution metrics (HU) and statistical analysis for the control group (repeated-measures ANOVA, FDR-adjusted).

**(a)**			
**Parameter**	**Estimate (CI 95%)**	***p*-Value**	**Interpretation**
Baseline HU (10 s)	146.5 (142.1–150.9)	—	Initial contrast distribution phase.
Peak HU (30 s)	245.5 (239.8–251.1)	<0.0001	Maximum contrast concentration in pulmonary artery.
Washout Rate (HU/s)	−1.73 (−1.95–−1.51)	<0.0001	Significant post-peak decline (~25% reduction).
Total AUC	10.50 (10.32–10.68)	—	Total area under the contrast–time curve.
**(b)**			
**Parameter**	**Estimate (CI 95%)**	***p*-Value**	**Interpretation**
Baseline HU (10 s)	139.0 (136.8–141.2)	—	Initial contrast distribution phase.
Peak HU (30 s)	239.1 (231.8–246.4)	<0.0001	Maximum contrast concentration in pulmonary artery.
Washout Rate (HU/s)	−1.96 (−2.24–−1.67)	<0.0001	Significant post-peak decline (~21% reduction).
Total AUC	10.00 (9.80–10.21)	—	Total area under the contrast–time curve.
**(c)**			
**Parameter**	**Estimate (CI 95%)**	***p*-Value**	**Interpretation**
Baseline HU (10 s)	216.7 (211.7–221.8)	—	Initial contrast distribution phase.
Peak HU (30 s)	310.3 (305.2–315.3)	<0.0001	Maximum contrast concentration in pulmonary artery.
Washout Rate (HU/s)	−6.39 HU/s (−5.62 to −7.1)	<0.0001	Significant post-peak decline (~60% reduction).
Total AUC	11.70 (11.49–11.91)	—	Total area under the contrast–time curve.

Note: Data are expressed as mean estimates with 95% confidence intervals (CIs). *p*-values adjusted for multiple comparisons using the Benjamini–Hochberg false discovery rate (FDR) correction. Statistical analysis was performed using repeated-measures ANOVA. AUC = area under the curve.

## Data Availability

The raw data supporting the conclusions of this article will be made available by the author on request.

## Abbreviations

The following abbreviations are used in this manuscript:

CTPA	Computed tomography pulmonary angiography
HU	Hounsfield Unit
SNR	Signal-to-noise ratio
CNR	Contrast-to-noise ratio
COPD	Chronic obstructive pulmonary disease
